# An evaluation of outpatient satisfaction in Chinese tertiary hospitals from a patient-centered perspective: a cross-sectional study

**DOI:** 10.3389/fpubh.2025.1698875

**Published:** 2026-01-12

**Authors:** Yutao Wei, Yongyi Xu, Dandan Chen, Yanling Fang, Kaitong Liang, Bo Deng, Lijun Ma, Yajun Yang, Qin Cao

**Affiliations:** 1 The Second Affiliated Hospital, Guangzhou Medical University, Guangzhou, China; 2 School of Business, Guangdong Polytechnic, Foshan, China; 3 School of Public Health and Management, Guangzhou University of Chinese Medicine, Guangzhou, China; 4 School of Health Sciences, University of Greenwich, London, United Kingdom

**Keywords:** doctor–patient relationship, factor analysis, outpatient satisfaction, patient-centered care (PCC), tertiary hospitals

## Abstract

**Background:**

As a key indicator of healthcare service quality in Chinese tertiary hospitals, patient satisfaction measurement requires multidimensional assessment. However, existing tools predominantly focus on clinical efficiency while neglecting humanistic dimensions such as emotional support and personalized care. This oversight renders them inadequate to address contemporary patients' growing expectations for holistic, patient-centered care (PCC) experiences.

**Objective:**

This study aims to develop a patient-centered evaluation system for measuring outpatient satisfaction in Chinese tertiary hospitals.

**Methods:**

We surveyed 2,664 patients from three outpatient departments of tertiary hospitals in Guangzhou using a validated self-developed questionnaire assessing overall satisfaction (satisfied/not satisfied) and four domains: outpatient environment, outpatient process, PCC, and hospital services. The sample was randomly and equally split for exploratory and confirmatory factor analysis (EFA/CFA) to identify and validate satisfaction dimensions. A weighted satisfaction score was derived, with model robustness tested through binary Logistic regression and Receiver Operating Characteristic (ROC) curve analysis.

**Results:**

Overall satisfaction was reported by 43.39% of respondents, with significant variation by age (χ^2^ = 10.615, *P* = 0.014) and inverse correlation with waiting time (χ^2^ = 44.989, *P* = 0.001). EFA results indicated good internal consistency (KMO = 0.912; Bartlett's test *P* < 0.01), with all four domains showing significant positive correlations (*P* < 0.01). PCC emerged as the strongest contributor (26.19%), followed by hospital services (17.62%), outpatient process (16.95%), and outpatient environment (8.79%). CFA revealed key indicators: environmental safety (SMC = 0.751), waiting order management (SMC = 0.820), physician-patient relationship (SMC = 0.929), and doctor's diagnosis and treatment (SMC = 0.819). The weighted satisfaction score significantly predicted overall satisfaction (*B* = 3.244, *P* < 0.01), with ROC analysis confirming good predictive validity (AUC = 0.771, *P* < 0.01).

**Conclusion:**

This study establishes the first validated patient-centered satisfaction evaluation system for Chinese outpatient care, identifying PCC and hospital services as key determinants of satisfaction. The findings provide an evidence-based framework for optimizing healthcare delivery and informing policy reforms to better address patients' evolving expectations in tertiary hospital settings.

## 1 Introduction

In China, tertiary hospitals represent the apex of the national hierarchical medical system. Equipped with advanced technologies and well-trained professionals, they provide high-level diagnosis, treatment, and healthcare service. As such, these institutions bear substantial responsibility for supporting public health within their regions, yet this critical role also renders them increasingly overwhelmed (1, 2). Outpatient departments, in particular, face challenges such as overcrowding, prolonged waiting times, and operational inefficiencies, contributing to declining service quality and patient satisfaction (3). In the past two decades, total healthcare expenditure has increased rapidly, thus the Chinese healthcare reform was introduced in 2009, the medical insurance institutions paid to relevant medical institutions in advance according to the number of patients they have, avoiding medical staff from prescribing expensive drugs and tests to patients for higher profit and rewards, the reduced patients' financial burden significantly improved patient satisfaction. Diagnosis-related group (DRG) payment models have been piloted nationwide since 2019 to reduce wasteful expenditures while improving service quality, whereas China's ongoing healthcare reform is needed to further improve quality of care and patient satisfaction (4). Moreover, rapid urbanization, population aging, and increasing cross-regional patient flows have intensified the strain on tertiary hospitals. These issues are further exacerbated by rising patient expectations for more efficient, convenient, and humanistic care, driven by the growth of Internet-based healthcare. Although the Internet hospital become a mainstream approach of health services and provide public welfare for patients during the epidemic, online service operates virtually, such that patients no longer receive tangible service as they would in hospital facilities and environments, which has led to a serious concern regarding patient satisfaction (5).

To address these issues, global research has significantly advanced the understanding and measurement of patient satisfaction, the USA, the UK, Germany, Sweden, Finland and Norway moved patients' experiences of treatment and satisfaction increasingly into the focus as key quality indicators and established routines measurement to improve the process of evaluating patient satisfaction and provide important feedback to the respective hospitals. For instance, the public U.K. healthcare system (NHS) appears to be currently leading the way in this regard not only because of the ongoing adaptation of their measurement tool, the National Health Service Inpatient Survey (NHSIP), but also because of the integration of traditional quantitative and Internet-based narrative feedback possibilities and their publication on the NHS website the USA introduced a Hospital Consumer Assessment of Healthcare Providers and Systems (HCAHPS) survey, which became the national standard for assessing patient satisfaction. Japan conducted nationwide satisfaction surveys by using the Japanese version of Primary Care Assessment Tool (JPCAT) (6). Compared to the countries with highly developed healthcare systems, Chinese government promulgated the Opinions on Strengthening the Performance Evaluation of Tertiary Public Hospitals in 2019 Since the “patient satisfaction” was included as an important indicator of the tertiary public hospitals' performance evaluation index system, research on outpatient satisfaction has gained significant attention (7, 8). Prior studies can be broadly categorized into two streams: those examining external influencing factors (e.g., demographics including gender, age, occupation, income, education level, marital status, and type of insurance, patients' cognition, and prior experiences) (9–11), and those developing evaluation frameworks, typically covering domains such as staff competence, facility environment, process efficiency, and cost (12–18). One factor that seems to be crucial for patient satisfaction across all these countries mentioned is patients' communication with physicians. On this aspect, patients in the U.K. seem to report the most positive satisfaction about their communication with healthcare professionals (6). However, existing tools often overlook the growing importance of patient-centered communication and humanistic aspects of care.

Recent policy developments, the Institute of Medicine defined Patient-Centered Care (PCC) as health care establishing a partnership between physicians and patients to ensure that decisions respect patients' wants, needs, and preferences and that patients are adequately educated and supported to make decisions and participate in their own care (19). A recent study that explores patient experience with PCC attached great importance to building a trusting relationship with physicians, open and honest communication, the physician gives a patient room to express preferences or ask questions. Moreover, PCC has previously been shown to be effective with significant improvements of patient satisfaction (20). In China, including the 2024 “Action Plan for Enhancing Medical Humanistic Care (2024–2027),” and 2025 “Reference Outline for the Medical Humanities Curriculum System in Medical Institutions,” underscore the national shift toward PCC (3). These growing policies emphasize empathy, communication, and respect for patient preferences The value of PCC is also widely acknowledged. For instance, all top performing medical systems worldwide promote PCC (21–23). Notably, Chinese healthcare system also has undergone rapid and profound changes to enhance patients' medical treatment experience and their perception of PCC (24, 25). However, the integration of PCC into satisfaction evaluation systems remains underdeveloped (26, 27). Most current tools lack empirical validation and do not adequately reflect the biopsychosocial dimensions of care (28).

To address these gaps, this study aims to develop and validate a reliable, multidimensional, patient-centered evaluation system tailored to the unique context of outpatient services in Chinese tertiary hospitals. The findings are intended to support evidence-based improvements in health service quality and patient experience.

## 2 Method

### 2.1 Study design and setting

This cross-sectional study was conducted from September to December 2023 as part of the Social Benefit Project of Public Health Institutions in Guangzhou—an initiative led by the Guangzhou Municipal Health Commission to enhance medical service quality and improve patient satisfaction. There are 15 tertiary general hospitals in this project, and they are all classified as third-grade hospitals according to the three-grade and 10-level management system for hospitals in China. A tertiary general hospital was selected from 15 hospitals in this project by using a simple random sampling design, which ensured a representative sample was achieved. This urban hospital located in Guangzhou, comprised 2,500 beds, with a staff of over 3,683 and an annual outpatient accounting of ~25,00,000, composed of three independent hospital branches, each of which has an independent outpatient department, contributing to an important position in the overall outpatient service due to high reputation and available technical expertise. Outpatients who agreed to participate were randomly selected for investigation in the outpatient department including first consultation or return visit, from more than 40 departments including internal, external, obstetrics and gynecology, otorhinolaryngology and so on. The ethical approval was granted by the Clinical Research and Application Ethics Committee of the Second Affiliated Hospital of Guangzhou Medical University (Approval Reference Number: 2023-ks-31).

Participation in the study was voluntary. Informed consent was obtained from all participants prior to data collection, the questionnaire was administered to outpatients through both face-to-face and online platforms (including SMS-based messages and calls). Verbal informed consent was obtained from the majority of participants (~90%), who completed the questionnaire online; for those who completed the questionnaire face-to-face (~10%), written informed consent was obtained.

Referred from another country study, the required sample size was estimated using the following formula (29):


$$\begin{array}{l} N=\frac{Z^{2}\times \left(p\times \left(1-p\right)\right)}{E^{2}} \end{array}$$

*N* is the sample size, *Z* is the *Z*-score corresponding to the desired confidence level (1.96 for 95% confidence level), *p* is the estimated proportion (set at 0.5 to sample size), and *E* is the acceptable margin of error (set at 0.05). the value of 0.5 for *P*, 0.05 for *E* were used as a conservative assumption because it maximizes the required sample size when there is no reliable prior estimate of the population proportion or variance. The initial calculation yielded a minimum sample size of 385. After adjusting for anticipated 10% non-response rate, the final target minimum sample size increased to 424. Further accounting for an expected valid response rate, a total of 2,800 questionnaires were distributed. After excluding invalid, incomplete, and duplicate responses, 2,664 valid questionnaires were retained at last. Based on the average daily number of outpatient visits in this hospital, ~20% of outpatients were included in the final analysis.

### 2.2 Research instrument

The questionnaire comprised three distinct sections:

- Informed consent and introduction: this section outlined the study's purpose, assured participants of data confidentiality, and obtained their consent.
- Demographic and visit-related information: participants were asked to provide background and consultation details, including age group, gender, educational level, payment method, waiting time, patient origin (local, within province, or out of province), and overall satisfaction (satisfied or unsatisfied).
- Outpatient satisfaction survey: this section evaluated four key domains of patient satisfaction: outpatient environment, outpatient process, PCC, and hospital services (see Table 1 for full details). These domains were developed based on input from experts participating in the Guangzhou Social Benefit Project for public medical institutions. The experts included the professors at School of Public Health, Shanghai Jiao Tong University and the directors of the outpatient departments of the First, Second, Third Affiliated Hospital of Guangzhou Medical University, Guangzhou First People's Hospital and Guangzhou Red Cross Hospital, who had rich experience in the management of outpatient. Each item was rated on a five-point Likert scale ranging from 1 (very unsatisfied) to 5 (very satisfied).

**Table 1:** Key variables, observed variables and observed description of outpatient satisfaction.

**Key domain (latent variable)**	**Item (observed variable)**
Outpatient environment	A1 Environmental safety
	A2 Facility amenities (e.g., chairs, wheelchairs, water dispensers)
	A3 Information transparency
Outpatient process	B1 Ease of registration process
	B2 Order in waiting area
	B3 Consultation duration (time from registration to seeing the doctor)
	B4 Waiting time
PCC	C1 Exploring disease experience (e.g., Whether the doctor gave sufficient time to describe the reason for your visit)
	C2 Communication clarity (e.g., Whether the doctor responded in an understandable manner)
	C3 Building doctor–patient rapport (e.g., Whether the doctor took time to answer your questions patiently)
	C4 Seeking shared understanding (e.g., Whether the doctor provided all necessary medical information)
	C5 Respecting patient preference (e.g., Whether the treatment plan respected your personal preferences)
Hospital services	D1 Nursing services
	D2 Doctor's diagnosis and treatment
	D3 Other staff services
	D4 Accessibility of medical reports
	D5 Digital service utility

Each domain included multiple specific indicators:

- Outpatient environment was assessed through three items: environmental safety, facility amenities, and information transparency.
- Outpatient process included four items: ease of registration, order in the waiting area, waiting time, and consultation duration.
- PCC was evaluated using five items: exploring disease experience, communication clarity, building doctor-patient rapport, seeking shared understanding, and respecting patient preferences.
- Hospital services consisted of five items: nursing services, doctor's diagnosis and treatment, other staff services, accessibility of medical reports and digital service utility.

### 2.3 Statistical analysis

#### 2.3.1 Identification of factors influencing outpatient satisfaction

Overall satisfaction (dichotomized as satisfied or unsatisfied) served as the dependent variable. Independent variables included age group, gender, educational level, payment method, consultation duration, and patient origin. Chi-square tests were performed using SPSS 25.0 to identify statistically significant factors. Variables with significant associations were subsequently included in a binary Logistic regression model for further analysis.

#### 2.3.2 Development of the outpatient satisfaction evaluation system

The total sample (*n* = 2,664) was randomly divided into two equal subsamples for EFA and CFA:

- Subsample 1 (*n* = 1,332) was used for EFA in SPSS 25.0. Internal consistency was assessed using Cronbach's alpha, and the validity was verified with the Kaiser-Meyer-Olkin (KMO) test and Bartlett's test of sphericity. Principal component analysis with varimax rotation was applied to extract factors, retaining factors with eigenvalues >0.8. The maximum number of iterations was set to 25. The resulting factor structure then informed the initial satisfaction model.
- Subsample 2 (*n* = 1,332) was used for CFA in AMOS 24.0. The Maximum Likelihood (ML) method was employed to estimate model parameters. Model fit was evaluated using three types of indices: Preliminary Fit Criteria (PFC), Overall Model Fit (OMF; e.g., χ^2^/df, CFI, RMSEA), and Fit of Internal Structure Model (FISM). Factor loadings and path coefficients were examined to assess relationships between latent and observed variables. The model was iteratively refined by releasing constrained parameters to improve fit. Correlations among latent variables were examined to evaluate higher-order factor structures.
- Weighting and validation: factor weights were derived from the variance contribution rates of each domain in the EFA results. A total satisfaction score was computed using a weighted formula. The score's discriminative power was tested against the binary measure of overall satisfaction using binary Logistic regression and Receiver Operating Characteristic (ROC) curve analysis in SPSS 25.0.

## 3 Results

### 3.1 Demographic characteristics of respondents

The effective sample size of this study is *n* = 2,664, the observed variables are of this questionnaire 17, and the variable-sample ratio is 156.7: 1, which meets the maximum requirements (EFA: 20:1, CFA:15:1). The unweighted total score of this questionnaire ranges from 17 to 85. Valid responses (2,664) fit a normal distribution with a mean (Standard Deviation) of 73.11 (9.27), a minimum of 20 and a maximum of 85 (unweighted).

Among the 2,664 valid outpatient responses, the majority were contributed by Hospital branch 1 (*n* = 1,867; 70.09%), followed by Hospital branch 2 (*n* = 734; 27.55%), and Hospital branch 3 (*n* = 63; 2.36%). Female participants accounted for 58.2% of the sample. 75.6% of patients were between 21 and 60 years of age and 69.3% held an educational qualification below a bachelor's degree. 63.2% of payment method was resident medical insurance. 86.1% of patients completed their visit within 2 h, and 71.2% were local residents of Guangzhou. Overall, 43.4% of respondents reported being satisfied with their outpatient experience. A detailed summary of participant characteristics is provided in Table 2.

**Table 2:** The characteristic of participants in this study (*N* = 2,664).

**Characteristics**	**Categories**	***n* (%)**	**Satisfaction rate (%)**	**Chi-square**	***P*-value**
Gender	Female	1,551 (58.22%)	43.58	0.133	0.716
	Male	1,113 (41.78%)	44.29		
Age group	≤20 years	86 (3.23%)	40.69	10.615	0.014
	21–40 years	1,146 (43.02%)	40.83		
	41–60 years	867 (32.55%)	47.98		
	>60 years	565 (21.21%)	44.24		
Education level	Below bachelor's degree	1,845 (69.26%)	44.66	2.174	0.337
	Bachelor's degree	695 (26.09%)	42.73		
	Master's or higher	124 (4.65%)	38.71		
Payment method	Self-funded	729 (27.36%)	41.01	4.160	0.125
	Government-funded	250 (9.38%)	47.60		
	Resident medical insurance	1,685 (63.25%)	44.56		
Waiting time	≤1 h	1,036 (38.89%)	50.67	44.989	0.001
	1–2 h	1,259 (47.26%)	41.93		
	2–3 h	281 (10.55%)	32.38		
	>3 h	88 (3.30%)	28.41		
Patient origin	Local (Guangzhou)	1,896 (71.17%)	44.93	3.116	0.211
	Within Province	515 (19.33%)	41.74		
	Out-of-Province	253 (9.50%)	40.32		

### 3.2 Differences in satisfaction among patient subgroups

Univariate analysis revealed statistically significant differences between overall satisfaction and both age group (χ^2^ = 10.615, *P* = 0.014) and waiting times (χ^2^ = 44.989, *P* = 0.001). No other demographic variables demonstrated significant associations with satisfaction (all *P* > 0.05).

A binary logistic regression model was subsequently constructed with overall satisfaction as the dependent variable, and age group and waiting time as independent variables (Tables 3, 4). The model demonstrated good fit, as confirmed by Hosmer–Lemeshow test's result (*P* > 0.05).

**Table 3:** Variables assignment of logistic regression model of overall satisfaction.

**Variables**	**Assignment**
Outpatient satisfaction	Satisfied = 1, unsatisfied = 0
Age group (years)	≤20 = 1, 21–40 = 2, 41–60 = 3, >60 = 4
Waiting time (hours)	≤1 = 1, 1–2 = 2, 2–3 = 3, >3 = 4

**Table 4:** The results of logistic regression model of overall satisfaction.

**Characteristic**	***P*-value**	**β**	**OR**	**95% CI lower**	**95% CI upper**
Age ≤ 20	0.568	−0.135	0.873	0.549	1.391
Age 21–40	0.521	−0.068	0.935	0.761	1.149
Age 41–60	0.048	0.218	1.243	1.002	1.542
Age >60 (ref)	–	–	–	–	–
Waiting ≤ 1 h	0.001	0.956	2.602	1.609	4.207
Waiting 1–2 h	0.015	0.591	1.806	1.120	2.912
Waiting 2–3 h	0.476	0.192	1.211	0.715	2.053
Waiting >3 h (ref)	–	–	–	–	–
Constant	0.001	−0.961	0.382	–	–

Regression results indicated that patients aged 41–60 years were significantly more likely to be satisfied than those over 60 years of age (OR = 1.243, β = 0.218, *P* = 0.048). Regarding waiting time, patients who waited ≤ 1 h (OR = 2.602, β = 0.956, *P* = 0.001) or 1–2 h (OR = 1.806, β = 0.591, *P* = 0.015) were significantly more likely to report satisfaction than those waiting >3 h. No significant difference in satisfaction was observed between patients waiting 2–3 h and those waiting >3 h.

### 3.3 Results of exploratory factor analysis (EFA)

The KMO test yielded a high value of 0.912 (normal range >0.60) for the outpatient satisfaction survey items, and Bartlett's test of sphericity was statistically significant (*P* < 0.01), supporting the factorability of the data. Internal consistency was confirmed with a Cronbach's alpha (total scale) of 0.855 (favorable >0.80, appropriate >0.70, acceptable >0.60), the four subscales were 0.646, 0.779, 0.872, 0.721, respectively (Table 5). Although the Cronbach's alpha of outpatient environment was not high, partly due to the quantity of items (*n* = 3) was less. The subscale was developed on the basis of both expert interview and previous reviews, and the total scale and the other three subscales were considered to fully cover key aspects of the core concept of outpatient satisfaction. Therefore, good internal consistencies of this measurement model was confirmed.

**Table 5:** The Cronbach's alpha of the key domains.

**Key domain (sub scale)**	***n***	**Cronbach's alpha**
Outpatient environment	3	0.646
Outpatient process	4	0.779
PCC	5	0.872
Hospital services	5	0.721

*n*, the quantity of items of key domain.

**Figure 1:**
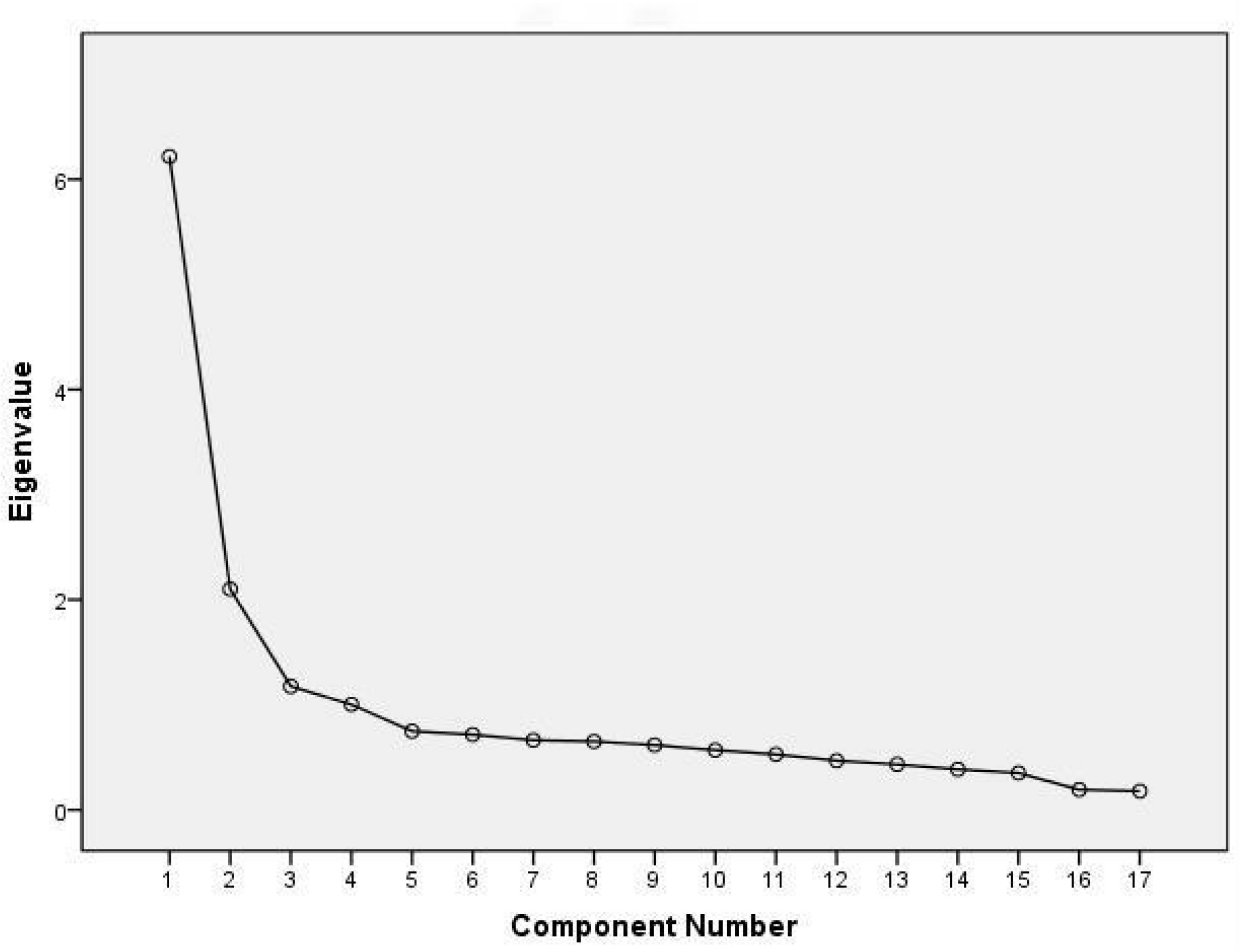
The scree plot of initial model of outpatient satisfaction evaluation system.

Figure 1 suggested a four-components solution. After seven iterations of rotation, a clear factor structure emerged from the rotated component matrix. The four extracted components were identified as: PCC, Hospital Services, Outpatient Process, and Outpatient Environment. These components accounted for 61.745% of the total variance, demonstrating strong explanatory power for the underlying structure of the measured variables (Table 6).

**Table 6:** The rotated component matrix of the initial model of outpatient satisfaction evaluation system.

**Observed variable**	**Component 1**	**Component 2**	**Component 3**	**Component 4**
	**PCC**	**Hospital services**	**Outpatient process**	**Outpatient environment**
A1				0.558
A2				0.581
A3				0.699
B1			0.641	
B2			0.586	
B3			0.709	
B4			0.721	
C1	0.862			
C2	0.875			
C3	0.878			
C4	0.649			
C5	0.720			
D1		0.581		
D2		0.786		
D3		0.730		
D4		0.737		
D5		0.463		
Eigenvalue	6.217	2.101	1.176	1.003
% of the variance	21.930	15.086	14.095	10.633
% of the accumulative variance	21.930	37.016	51.111	61.745

### 3.4 Confirmatory factor analysis (CFA) results

#### 3.4.1 Initial model

**Figure 2:**
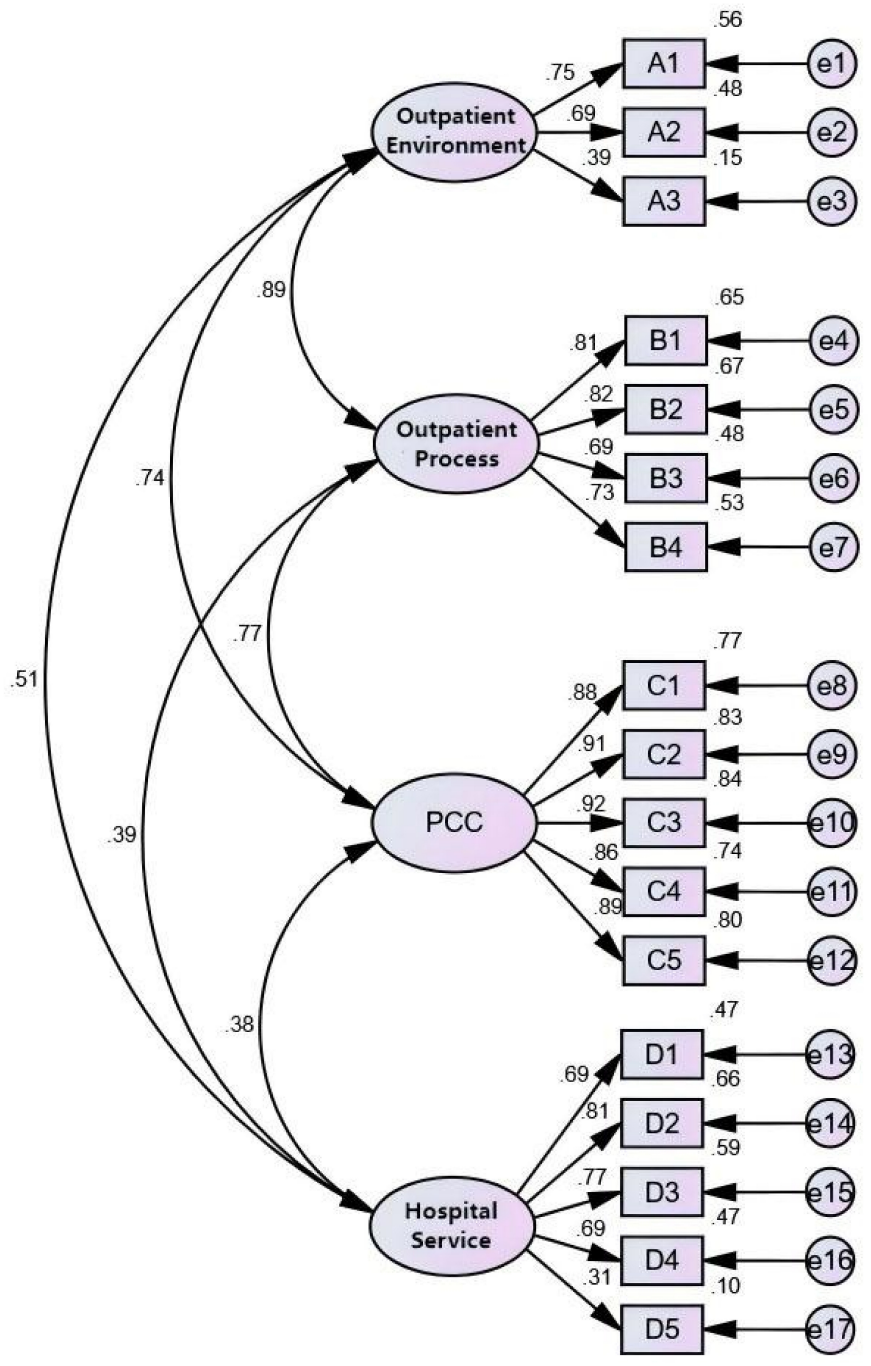
CFA and SRW of initial model of outpatient satisfaction evaluation system. The lines represent corresponding SRW values that are statistically significant, meaning that *P* < 0.05.

Sub-sample 2 was used to validate the initial model derived from the EFA and to estimate its parameters. The OMF met acceptable standards: the Goodness-of-Fit Index (GFI), Adjusted Goodness-of-Fit Index (AGFI), Root Mean Square Error of Approximation (RMSEA), Normed Fit Index (NFI), Comparative Fit Index (CFI), Incremental Fit Index (IFI), Tucker–Lewis Index (TLI), Parsimony Goodness-of-Fit Index (PGFI), Parsimony Normed Fit Index (PNFI), and the ratio of Chi-square to degrees of freedom (CMIN/DF) all fell within recommended thresholds [(30, 31); Table 7]. As shown in Figure 2, the FISM was also satisfactory, as all path coefficients between latent variables were directionally consistent with theoretical expectations and statistically significant (*P* < 0.01). However, the PFC were not fully met. Specifically, the standardized regression weights (SRWs) for “A3 → Outpatient Environment” and “D5 → Hospital Services” were 0.39 and 0.31, respectively, falling below the conventional threshold of 0.5 (Table 8). These results suggest that the initial model did not align adequately with the observed data, indicating a need for refinement. To avoid overfitting and ensure clinically meaningful adjustments, subsequent modifications were guided not only by statistical indications but also by theoretical relevance and practical considerations in clinical contexts (32).

**Table 7:** The fit indices of initial and modified model of outpatient satisfaction evaluation system.

**Statistical index**		**Recommended threshold**	**Initial model**	**Modified model**
AFI	RMSEA	<0.08	0.052	0.038
	GFI	>0.9	0.956	0.978
	AGFI	>0.9	0.940	0.967
IFI	NFI	>0.9	0.963	0.983
	TLI	>0.9	0.965	0.985
	RFI	>0.9	0.955	0.977
	IFI	>0.9	0.971	0.989
	CFI	>0.9	0.971	0.989
PFI	CMIN/DF	<5 appropriate, <3 favorable	4.588	2.949
	PNFI	>0.5	0.800	0.740
	PGFI	>0.5	0.706	0.644

**Table 8:** The CFA results of initial model of outpatient satisfaction evaluation system.

**Latent variable**	**Observed variable**	***S.E*.**	***P*-value**	**SRW**	**CR**	**AVE**
Outpatient environment	A1 (ref)			0.747	0.648	0.395
	A2	0.044	<0.001	0.691		
	A3	0.079	<0.001	0.390		
Outpatient process	B1 (ref)			0.809	0.848	0.584
	B2	0.03	<0.001	0.818		
	B3	0.039	<0.001	0.692		
	B4	0.035	<0.001	0.731		
PCC	C1 (ref)			0.878	0.951	0.796
	C2	0.02	<0.001	0.912		
	C3	0.021	<0.001	0.918		
	C4	0.024	<0.001	0.860		
	C5	0.021	<0.001	0.894		
Hospital service	D1 (ref)			0.688	0.797	0.458
	D2	0.052	<0.001	0.815		
	D3	0.046	<0.001	0.766		
	D4	0.052	<0.001	0.686		
	D5	0.086	<0.001	0.311		

#### 3.4.2 Modified model

Two items with low SRWs were identified and removed to improve model fit. Item A3 (“information transparency”) showed a weak association with the latent variable “Outpatient Environment,” suggesting that patients may have placed relatively low importance on public information dissemination in the hospital settings studied. Similarly, item D5 (“digital service utility”) loaded poorly on “Hospital Services,” reflecting the still-limited adoption and utilization of internet-based hospital services compared to traditional face-to-face consultations.

**Figure 3:**
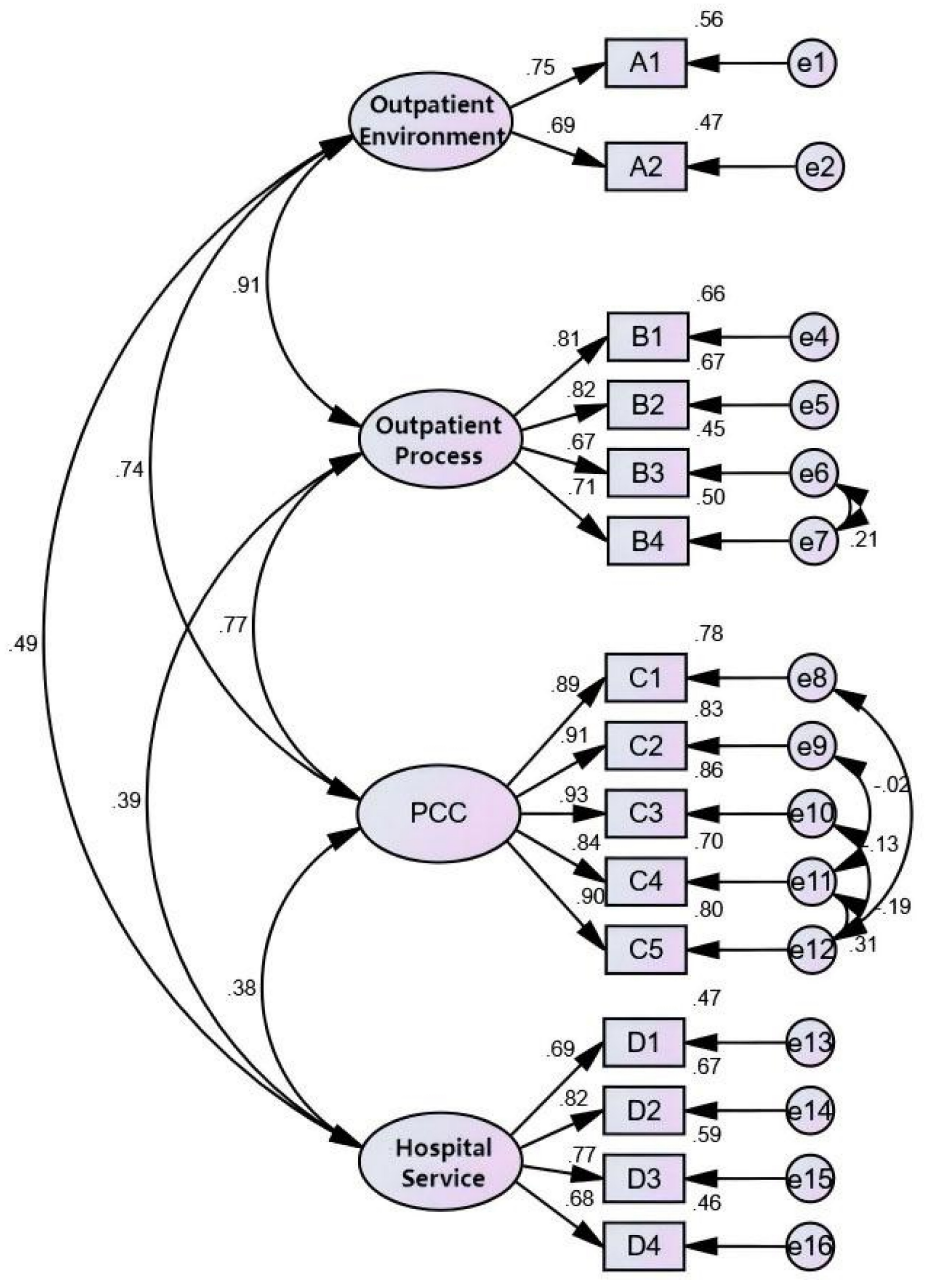
CFA and SRW of modified model of outpatient satisfaction evaluation system. The lines represent corresponding SRW values that are statistically significant, meaning that *P* < 0.05.

Following the removal of A3 and D5, the CFA model was re-estimated. To further enhance model fit, covariances between error terms were introduced based on modification indices (MIs), in line with the procedure recommended by Thakkar (33). Specifically, covariances were added between the following pairs of error terms: “e11 <->e12,” “e6 <->e7,” “e9 <->e11,” “e10 <->e12,” and “e8 <->e12,” all of which exhibited high MI values. These adjustments resulted in a modified model (Figure 3) that showed marked improvement across all fit criteria. In Table 9, all PFC were satisfied, with SRWs exceeding 0.5. OMF indices also improved, including a CMIN/DF ratio below 3, indicating good fit. The FISM was also enhanced. They collectively supporting the soundness and robustness of the final model.

**Table 9:** The CFA results of modified model of outpatient satisfaction evaluation system.

**Latent variable**	**Observed variable**	***S.E*.**	***P*-value**	**SRW**	**CR**	**AVE**
Outpatient environment	A1 (ref)			0.751	0.681	0.517
	A2	0.044	<0.001	0.686		
Outpatient process	B1 (ref)			0.815	0.841	0.572
	B2	0.03	<0.001	0.820		
	B3	0.039	<0.001	0.669		
	B4	0.035	<0.001	0.710		
PCC	C1 (ref)			0.885	0.951	0.796
	C2	0.02	<0.001	0.912		
	C3	0.02	<0.001	0.929		
	C4	0.024	<0.001	0.840		
	C5	0.023	<0.001	0.895		
Hospital service	D1 (ref)			0.687	0.828	0.549
	D2	0.053	<0.001	0.819		
	D3	0.046	<0.001	0.768		
	D4	0.052	<0.001	0.681		

Correlation analysis revealed significant positive relationships among the four key latent variables (Table 10). Strong correlations were observed among “Outpatient Environment,” “Outpatient Process,” and “PCC” (*R* = 0.906, 0.767, 0.742, respectively), while “Hospital Services” was moderately correlated with the other three (*R* = 0.489, 0.395, 0.377). The SRWs also highlighted the relative contribution of each observed variable to its respective latent construct: “Environmental Safety” (square multiple correlation, SMC = 0.751) was the strongest indicator of “Outpatient Environment”; “Waiting Order” (SMC = 0.820) for “Outpatient Process”; “Enhancing the Doctor–Patient Relationship” (SMC = 0.929) for “PCC”; and “Technical Services” (SMC = 0.819) for “Hospital Services.”

**Table 10:** The results of correlation analysis of four key latent variables.

**Latent variable**	**Outpatient environment**	**Outpatient process**	**PCC**	**Hospital service**
Outpatient environment	/			
Outpatient process	0.906^**^	/		
PCC	0.742^**^	0.767^**^	/	
Hospital service	0.489^**^	0.395^**^	0.377^**^	/

^**^P < 0.01.

### 3.5 Calculation of the total score

**Figure 4:**
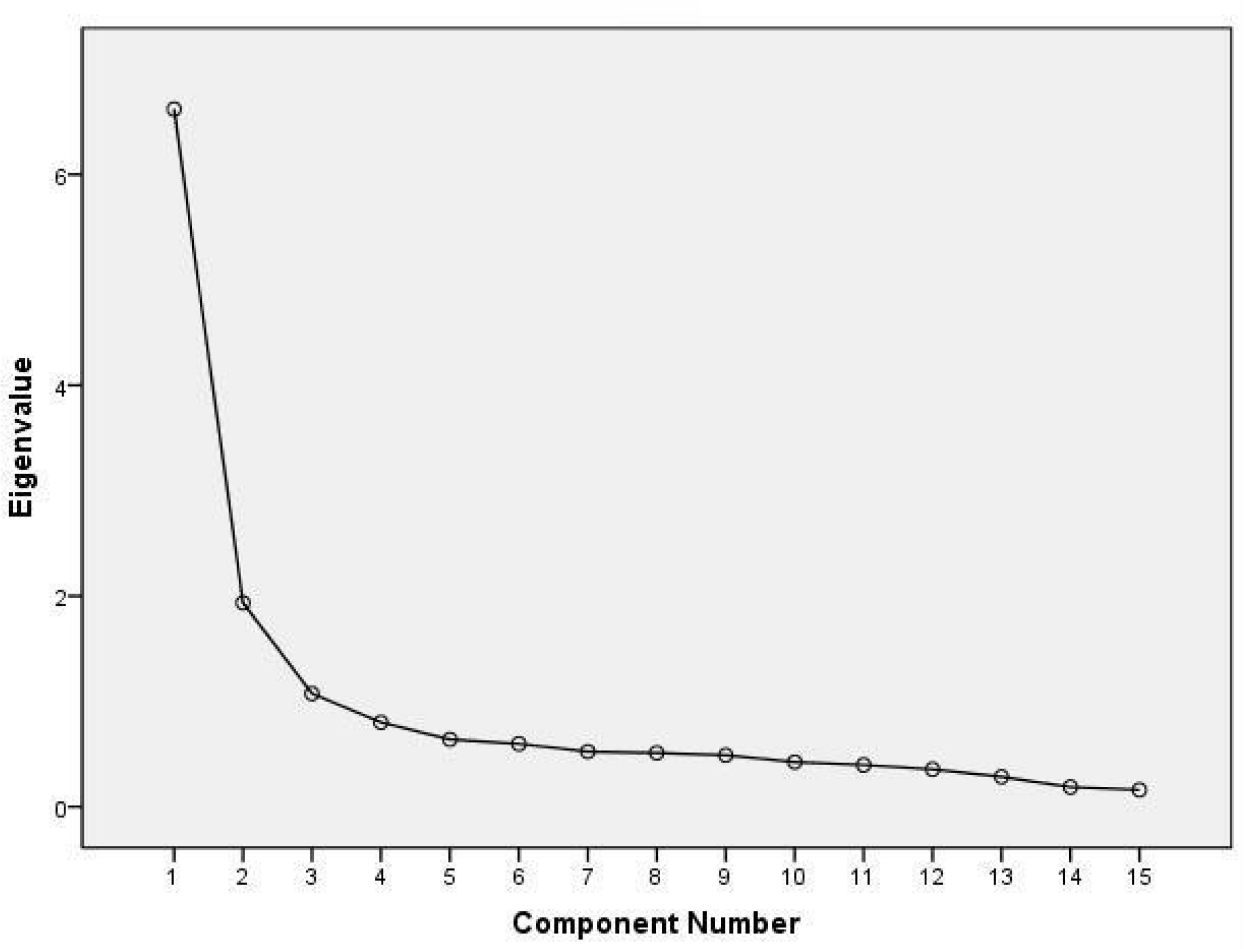
The scree plot of modified model of outpatient satisfaction evaluation system.

Based on the modified model validated by both EFA and CFA, the four latent variables—Outpatient Environment, Outpatient Process, PCC, and Hospital Services—were retained as core dimensions of patient satisfaction. Using the full dataset (*n* = 2,664), we conducted EFA to estimate the variance contribution of each component, which was subsequently used as its weight in the composite satisfaction score. As shown in Table 11 and Figure 4, the weights were assigned as follows:

- PCC (*W*1) = 26.186%
- Hospital services (*W*_2_) = 17.619%
- Outpatient process (*W*3) = 16.945%
- Outpatient environment (*W*4) = 8.794%

**Table 11:** The rotated component matrix of the modified model of outpatient satisfaction evaluation system.

**Observed variable**	**Component 1**	**Component 2**	**Component 3**	**Component 4**
	**PCC**	**Hospital services**	**Outpatient process**	**Outpatient environment**
A1				0.564
A2				0.835
B1		0.657		
B2		0.672		
B3		0.752		
B4		0.751		
C1	0.849			
C2	0.867			
C3	0.871			
C4	0.731			
C5	0.775			
D1			0.661	
D2			0.833	
D3			0.765	
D4			0.756	
Eigenvalue	6.621	1.935	1.074	0.801
% of the variance	26.186	17.619	16.945	8.794
% of the accumulative variance	26.186	43.805	60.751	69.544

Together, these four components accounted for 69.544% of the total variance, a 7.79% increase compared to the initial model. It indicates substantially improved explanatory power.

The total score (TS) of satisfaction was computed as a weighted composite of the four latent factor scores using the following formula:


$$\begin{array}{l} TS=\overset{4}{\underset{n=1}{\sum }}Wn*Fn \end{array}$$

- *Wn* (*n* = 1, 2, 3, 4) is the weight coefficient of factor *n*
- *Fn* (*n* = 1, 2, 3, 4) is the standardized score of factor *n*.

### 3.6 Validation of model robustness

**Figure 5:**
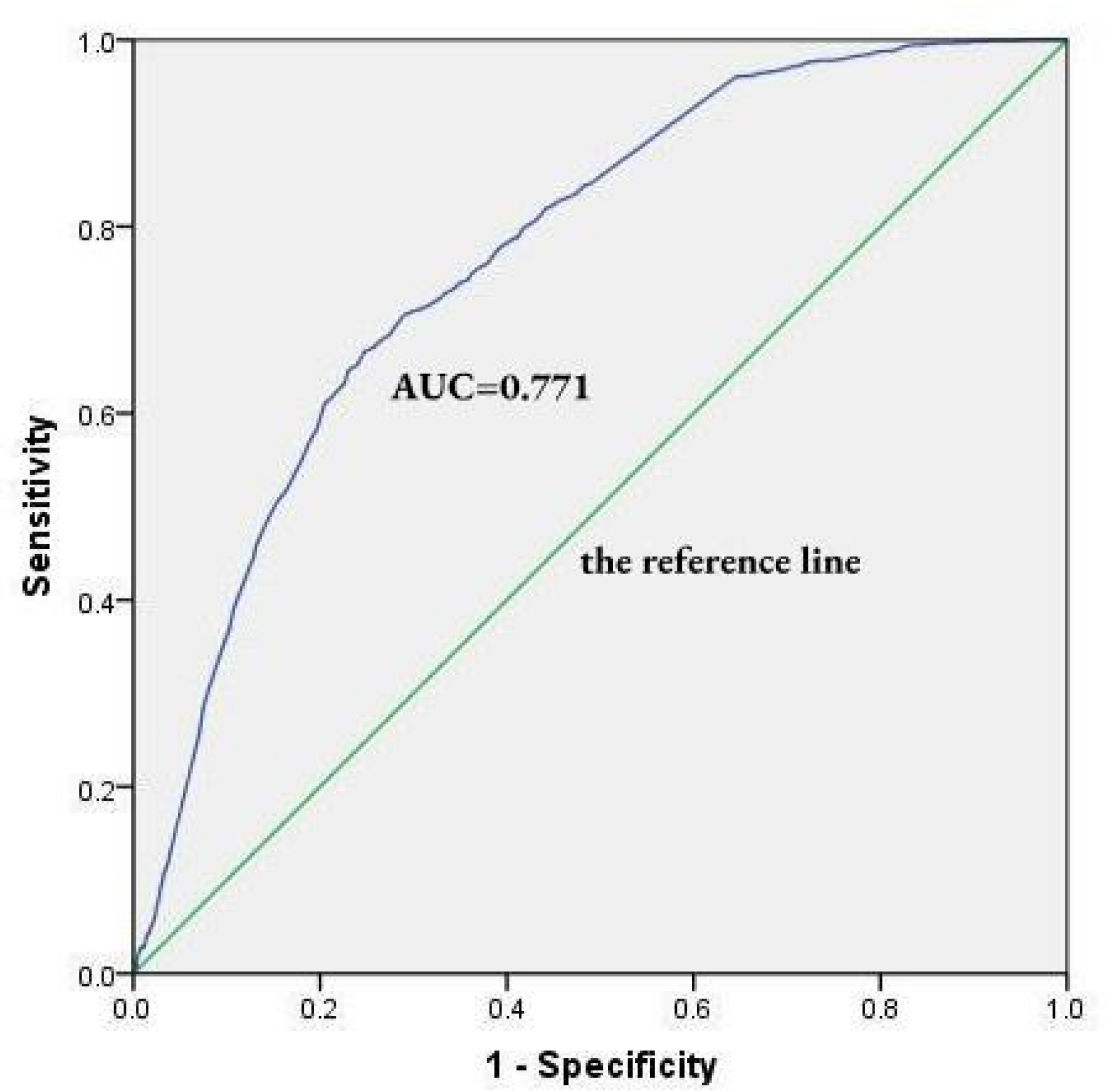
The ROC curve of modified model of outpatient satisfaction evaluation system.

To evaluate the robustness of the satisfaction evaluation model, binary Logistic regression and Receiver Operating Characteristic (ROC) curve analyses were performed. Overall satisfaction (coded as 1 for satisfied, 0 for unsatisfied) served as the dependent variable (*Y*), and the TS was entered as the independent variable (*X*). Logistic regression yielded a significant positive coefficient (Beta = 3.244, *P* < 0.01), indicating that higher TS values were strongly associated with greater likelihood of satisfaction (the outcome tending toward 1, Table 12). To further assess the model's predictive performance, an ROC curve was generated (Figure 5), plotting sensitivity on the *Y*-axis and 1—specificity on the *X*-axis. The curve lay well above the reference line (the diagonal line, *y* = *x*), suggesting excellent discriminative ability. The area under the curve (AUC) was 0.771 (95% CI: 0.753–0.788, *P* < 0.01), confirming that the model performed significantly better than chance (AUC = 0.5). These results collectively support the model's strong predictive validity and its utility in identifying and evaluating outpatient satisfaction levels.

**Table 12:** Logistic regression results of the robustness of the satisfaction evaluation model.

**Variable**	**Beta**	**S.E**.	**Wald**	***P*-value**
*X*	3.244	0.153	446.795	<0.01
Constant	−0.348	0.045	60.381	<0.01

## 4 Discussion

Based on the substantial volume of outpatient data collected in Guangzhou, this study developed and validated a patient-centered evaluation system for outpatient satisfaction within Chinese tertiary hospitals. The system not only assessed current satisfaction level but also identified key influencing variables, providing an evidence-based foundation for improving outpatient experience and service quality.

Despite extensive research on outpatient satisfaction, a consensus on its definition and optimal measurement methodology remains elusive. Few studies have systematically examined its influencing mechanisms from a humanistic care perspective (34). This study developed a patient-centered evaluation system specifically for tertiary hospital outpatients in China, thereby addressing this research gap. While previous studies have predominantly relied on self-designed questionnaires—often using five-point Likert scales, and occasionally 3-, 4-, or 7-point variants—this study employed the five-point format for its optimal balance between reliability and respondent engagement. This approach avoids the excessive coarseness of shorter scales and reduces the cognitive burden associated with longer ones (e.g., seven-point scales). Furthermore, where prior research has largely focused on providers' professional competence and attitudes, physical facilities, and operational processes, sometimes adapting instruments such as the Inpatient Satisfaction Questionnaire (35), this study introduced PCC as a core dimension alongside three conventional variables: outpatient environment, process, and hospital services. The proposed system was rigorously validated through reliability analysis, construct validity testing, model-fit evaluation, and robustness checks, with the relative weights and structural relationships among key variables clearly delineated. In summary, compared to emerging and increasingly important sources of outpatient satisfaction, the additional and underrepresented dimension such as humanistic care that are not yet captured in the conventional questionnaires, our study integrated the domain of PCC into existing assessment measures, developed and validated a patient-centered satisfaction evaluation framework, supporting continued advancement toward more humanistic and responsive care systems. This, in turn, will enable policymakers to make more informed decisions aimed at improving the quality of the outpatient, ultimately advancing toward more humanistic and responsive care models.

Based on the four domains of outpatient satisfaction, firstly, our result identified PCC as the most influential factor. With evolving health expectations, non-clinical aspects of care, including respect, comfort, and confidentiality, are receiving increasing attention from patients. Thus, enhancing humanistic dimensions of healthcare is critical for improving satisfaction (36). This finding consistent with a growing body of literature linking PCC to biopsychosocial models of care that emphasize care coordination, continuity, prepared teams, and shared decision-making. These elements help mitigate limitations of traditional disease-centered approaches, restore humanistic values in medical practice, and improve both the quality and efficiency of health services (37, 38). Specifically, fostering the doctor–patient relationship emerged as the most important sub-dimension of PCC, corroborating the work of scholars such as Khanal, who found that doctor-patient concordance strengthens trust, improves recovery outcomes, and elevates perceived care quality (29). Trummer et al. (39) similarly highlighted that strong interpersonal relationships between doctors and patients reduce uncertainty and anxiety, thereby increasing satisfaction through tension reduction. Social exchange theory further supports this: when perceived benefits outweigh costs, individuals are more likely to value the relationship and engage in future collaboration (40). In outpatient contexts, patients seek respect, empathy, and participatory engagement, while clinicians benefit from improved adherence. A constructive doctor–patient relationship facilitates mutual goals and enhances both clinical experiences and outcomes (41).

Secondly, in addition to humanistic factors, clinical service quality—particularly physicians' technical competence—also significantly influenced satisfaction. This aligns with existing evidence that medical service quality exerts strong positive effects on patient satisfaction (34). Among healthcare providers, doctors had a greater impact than nurses or administrative staff, a finding consistent with studies such as Hu et al. (36) which identified physician service quality as the primary determinant of satisfaction. This emphasis reflects China's hierarchical medical system, which designates tertiary hospitals for complex conditions while promoting first-contact care at primary institutions (42). Studies show that Chinese patients prioritize clinical service quality above other aspects of care (43), with most outpatients seeking medical services primarily for curative purposes (44). However, due to perceived inadequacies in primary care quality, patients often bypass local facilities even for non-urgent conditions, seeking instead the advanced expertise of tertiary hospitals. When patients perceive that their health needs are met through high-quality diagnosis and treatment, their trust and satisfaction increase substantially.

Thirdly, outpatient process was directly associated with outpatient satisfaction as well, which was consistent with the regression result: longer waiting times significantly reduced satisfaction, particularly beyond 2 h, linking prolonged waits to patient frustration (45, 46). This situation is exacerbated by structural urban–rural health inequities that drive patients from underserved areas to seek care in tertiary hospitals, resulting in overcrowding, extended waits, and shorter consultations, which collectively diminish the care experience (36). In this study, ~30% of outpatients were not local residents, noticeably 20% were from other regions of Guangdong province, such as the western, eastern and northern region, this is for the medical development levels in Guangdong province vary across regions, which was similar to the unbalanced development in different regions of China. Although the Chinese government has introduced policies aimed at alleviating these pressures—such as the 2015 healthcare reform designed to establish a hierarchical medical system, strengthen primary care services, and reduce the burden on tertiary hospitals—the effects so far have been limited (47). Persistent challenges remain in rebalancing patient flows and equitably distributing medical resources across different regions and levels of care.

Lastly, outpatient environment occupied the minimum weight, it means that outpatients pay less attention to facilities and environment compared with the former three domains. Although the outpatients' satisfaction with the medical environment improve their medical experience, after being more familiar with hospital environment, patients tend to demand more additional services than environment (48). Another finding in our study reaffirmed the impact of demographic factor on satisfaction. As in other settings, older adults tended to report higher satisfaction than younger patients, though those over 60 were less satisfied than those aged 41–60, a pattern potentially attributable to digital healthcare barriers. Widespread adoption of online appointment systems, electronic payments, and self-service kiosks has inadvertently disadvantaged older adults who may lack digital literacy or access, reducing their satisfaction with so-called “smart” hospital services.

This study has several notable strengths. First, it was conducted under the support of Guangzhou's Social Benefit Project for public healthcare institutions, which facilitated access to substantial and reliable data sources. Second, the use of mixed-mode data collection—incorporating both face-to-face and online methods (e.g., SMS and telephone calls)—helped mitigate selection bias that can arise from homogeneous recruitment strategies. Third, the proposed model was validated using a large sample and multiple analytical techniques. To our knowledge, this is the first study to develop and validate a structured PCC based satisfaction evaluation framework tailored to tertiary hospital outpatients in China. Finally, moving beyond mere descriptive accounts of satisfaction levels, this study elucidated the relative importance and structural relationships among key variables, providing actionable insights for service improvement.

Several limitations should also be acknowledged. As a cross-sectional survey, no causal inferences between the variables could be made. In addition, all participants were recruited from tertiary hospitals within Guangzhou, which may limit the generalizability of the findings to other regions or healthcare settings. Furthermore, the exclusive use of quantitative methods restricted deeper exploration of participants' lived experiences or the subjective reasons underpinning their satisfaction or dissatisfaction.

Future research should pursue multi-regional studies to enhance the external validity of the findings. There is also a need to develop more comprehensive and psychometrically robust tools to evaluate outpatient satisfaction across diverse contexts in China. In particular, further investigation is warranted into the impact of digital health initiatives on patient experiences and satisfaction in outpatient settings.

## 5 Conclusion

This study developed and validated a novel patient-centered satisfaction evaluation framework for outpatients in Chinese tertiary hospitals. The findings reveal that PCC and hospital services serve as primary factors influencing satisfaction, while satisfaction levels also vary significantly according to patient age and waiting time. Based on these findings, our study provided some implications for hospital administrators and decision-makers to meet increased needs and satisfaction of patients. Firstly, we propose that tertiary hospitals should actively adapt healthcare services toward more patient-centered. Training programs are valuable for supporting medical staff to develop communication skills grounded in empathy and clarity, using understandable language to explain complex terms, allowing sufficient time for questions, and responding attentively to more patients' emotional and clinical needs, these efforts are essential for building a good relationship between healthcare service providers and patients. Secondly, to address the challenge regarding the overwhelming caseloads, tertiary hospitals should not only strengthen the construction of amenities and optimize triage of the outpatients, but also improve the outpatient appointment service and rationalizing the visit schedule of patients may also shorten their waiting time, improve outpatient satisfaction. On the other hand, we argued simply expanding the capacity of tertiary hospitals or increasing staff numbers alone is not a sustainable solution in the current healthcare system. Instead, more preferential policies and financial support should be given to primary medical institutions to address these problems. Policy makers should further optimize the efficiency of medical resource allocation, improve the service capabilities of primary care institutions by equipping them with adequate and competent medical professionals, including general practitioners. Such systemic improvements would help divert patient flow from tertiary hospitals and alleviate the problems of overcrowding.

## Data Availability

The raw data supporting the conclusions of this article will be made available by the authors, without undue reservation.
